# Severe but not moderate hyperoxia of newborn mice causes an emphysematous lung phenotype in adulthood without persisting oxidative stress and inflammation

**DOI:** 10.1186/s12890-019-0993-5

**Published:** 2019-12-16

**Authors:** Anke Kindermann, Leonore Binder, Jan Baier, Beate Gündel, Andreas Simm, Roland Haase, Babett Bartling

**Affiliations:** 1 Department of Cardiac Surgery, Middle German Heart Center, University Hospital Halle (Saale), Martin Luther University Halle-Wittenberg, Ernst-Grube-Str. 40, 06120 Halle (Saale), Germany; 2 Department of Neonatology and Pediatric Intensive Care, Clinic for Child and Adolescent Medicine, University Hospital Halle (Saale), Martin Luther University Halle-Wittenberg, Halle (Saale), Germany

## Abstract

**Background:**

Preterm newborns typically require supplemental oxygen but hyperoxic conditions also damage the premature lung. Oxygen-induced lung damages are mainly studied in newborn mouse models using oxygen concentrations above 75% and looking at short-term effects. Therefore, we aimed at the investigation of long-term effects and their dependency on different oxygen concentrations.

**Methods:**

Newborn mice were exposed to moderate vs. severe hyperoxic air conditions (50 vs. 75% O_2_) for 14 days followed by a longer period of normoxic conditions. Lung-related parameters were collected at an age of 60 or 120 days.

**Results:**

Severe hyperoxia caused lower alveolar density, enlargement of parenchymal air spaces and fragmented elastic fibers as well as higher lung compliance with peak airflow limitations and higher sensitivity to ventilation-mediated damages in later life. However, these long-term lung structural and functional changes did not restrict the voluntary physical activity. Also, they were not accompanied by ongoing inflammatory processes, increased formation of reactive oxygen species (ROS) or altered expressions of antioxidant enzymes (superoxide dismutases, catalase) and lung elasticity-relevant proteins (elastin, pro-surfactant proteins) in adulthood. In contrast to severe hyperoxia, moderate hyperoxia was less lung damaging but also not free of long-term effects (higher lung compliance without peak airflow limitations, increased ROS formation).

**Conclusions:**

Severe but not moderate neonatal hyperoxia causes emphysematous lungs without persisting oxidative stress and inflammation in adulthood. As the existing fragmentation of the elastic fibers seems to play a pivotal role, it indicates the usefulness of elastin-protecting compounds in the reduction of long-term oxygen-related lung damages.

## Background

The respiratory management of preterm newborns includes supplemental oxygen because the immature lung is unable to maintain sufficient gas exchange [1]. However, the supplemental oxygen therapy using hyperoxic gas can also lead to hyperoxia-induced lung injury [2]. In the case of very preterm infants treated with high concentrations of oxygen, these severe hyperoxic conditions may contribute to bronchopulmonary dysplasia (BPD) as the combined result of lung immaturity and hyperoxia-mediated generation of reactive oxygen species (ROS) [3, 4]. Main characteristics of the BPD are the abnormal development of lung parenchyma, conducting airways and pulmonary vasculature that finally cause restrictions in gas exchange, airway hyperreactivity, pulmonary hypertension and, thus, lower physical capabilities in childhood and also later in life [5].

An increase in intra- and extra-cellular ROS due to the increased supply of oxygen and, therefore, increased alveolar oxygen concentration plays a pivotal role in mediating lung cellular damages [2]. Initially, hyperoxia causes the generation of superoxide anion (O_2_^•-^) molecules through the mitochondrial oxidative phosphorylation system in a higher amount than they can be simultaneously detoxified by dismutation to hydrogen peroxide [2]. Moreover, O_2_^•-^ is increasingly generated by members of the nicotinamide adenine dinucleotide phosphate (NADPH) oxidase (NOX) enzyme family at the outer cell membrane [2]. The O_2_^•-^ excess then causes the generation of other types of ROS molecules with subsequent cell damage through the oxidation of lipids and proteins, which again leads to secondary ROS generation by attracted immune cells [2]. In addition to the cell damaging effect, ROS act as cell signaling molecules supporting an aberrant generation of the lung extracellular matrix [6].

Newborn mice are commonly used as animal model to investigate cellular and molecular processes that contribute to the hyperoxia-mediated lung injury in preterm infants. In contrast to human alveolar development beginning prior to birth, murine alveolarization begins after birth on postnatal day (PND) 3, and the saccular phase of the lung development is already completed by PND15 [7]. Most mouse experimental studies were performed with high concentrations of oxygen (≥75% O_2_) from birth until PNDs 4–14 as they focused on the investigation of BPD development [8]. However, many of the preterm infants do not require comparably high oxygen concentrations for treating the respiratory distress syndrome due to the established surfactant replacement therapy and other improvements in their respiratory management [1]. In addition to the relatively high oxygen concentrations applied in experimental settings, most mouse studies assessed the effect of neonatal hyperoxia on lung structure and function in young or young-adult animals (PNDs 7–56) but, except for few cases [9–13], not later in life. Therefore, there is still a shortage of studies investigating the respiratory system of adult mice (>PND56) which were exposed to less severe hyperoxic conditions as newborns.

Another disadvantage of existing mouse experimental studies is the lack of lung functional analyses recording especially the peak expiratory flows, in addition to compliance and airway resistance, in order to better assess the elastic recoil of the lung tissues. This would be of high importance as clinical investigations showed lower forced expiratory volumes in 1 s (FEV_1_) and/or forced expiratory flow rates at 25–75% vital capacity (FEF_25–75%_) in young-adult survivors of BPD [14, 15], and a correlation of expiratory limitations and lung structural changes indicating the development of emphysema [16, 17].

Surprisingly low is also the number of mouse experimental studies assessing long-term oxidative damages of lung proteins [18, 19], and no study determined the current rate of lung ROS formation in adulthood. As we supposed similarities in the persistent lung changes due to neonatal hyperoxia between human and mouse, our study aimed at the identification of emphysema-like lung phenotype and its possible relation to oxidative stress-related parameters in adult mice exposed as newborns to moderate vs. severe hyperoxic air conditions (50 vs. 75% O_2_).

## Methods

### Study design

Newborn mice of the C57BL/6 N strain (Charles River, Sulzfeld, Germany) were treated from birth until PND14 with different concentrations of oxygen to reach normoxic (N; 21% O_2_), moderate hyperoxic (mH; 50% O_2_) and severe hyperoxic (sH; 75% O_2_) normobaric air conditions. This study was approved by the local Commission for Animal Protection. Hyperoxic air was created in a polycarbonate chamber equipped with computerized gas flow control units (Aera FC-R7800; Hitachi Metals, Obuke, Japan) and O_2_ gas detector (GfG, Dortmund, Germany), which was large enough for three standard cages. Maximum six newborns of each sex per lactating dam were exposed to either N or mH conditions in one experimental cycle or they were exposed to either N or sH conditions in another experimental cycle. Lactating dams were rotated between hyperoxic and normoxic conditions every day to minimize maternal effects. Because of this experimental procedure, our study generated more mice of the N than mH or sH group. Analyses were performed at the PNDs 14, 60 and/or 120. At PND30, 17 female mice each treatment group were individually placed in a standard cage equipped with a running wheel until PND60. Wheel-running activity (PNDs 53–60) was recorded by use of a data acquisition unit connected to the wheel-running unit as described [20]. In addition, we used newborn (PND0) and PND280 mice for the analyses of expression, ROS and lung function. Because of lung structural and functional analysis at different ages, which were not possible by use of the same mouse, the precise number of mice used each group (minimum 12 mice) is given in the legends of tables and figures, respectively.

Pregnant dams were always housed at specific pathogen-free conditions. After giving birth, dames and their newborns were transferred into an experimental room equipped with oxygen chamber and wheel running system, and housed at conventional conditions (21 °C, 45% humidity, 12-h light/dark cycle, ad libitum access to water and food). Newborns were marked by foot tattoos for their identification. General health and body weights of the experimental mice were checked every day till PND15, every 2 days between PNDs 16–30, and every week after PND31. At the PND of analysis, mice were killed by cervical dislocation, and lung and blood were prepared for further analysis.

### Analysis of bronchoalveolar lavage (BAL) and blood

A bronchoalveolar lavage (BAL) was performed three times with 0.3 ml phosphate-buffered saline and then pooled. After counting the cell number, BAL cells were centrifuged on a slide and stained with the HemaDiff Quick Staining kit (Bioanalytic GmbH, Umkirch, Germany) for cytological investigations. Cell-free BAL fluid was obtained by centrifugation and analyzed for protein with the Pierce™ bicinchoninic acid (BCA) Protein Assay kit (Thermo Fisher Scientific, Rockford, IL), for IgM with the mouse IgM ELISA kit (Bethyl Laboratories, Montgomery, TX) and for soluble receptor for advanced glycation end-products (sRAGE) with the mouse RAGE DuoSet kit (R&D Systems, Minneapolis, MN). Heparinized blood was taken after transection of the vena cava and subjected to automated blood cell count using the scil Vet ABC™ Hematology Analyzer (scil animal care company GmbH, Viernheim, Germany).

### Lung histology

Lung histology was assessed by use of the left lung, which was fixed with 4% phosphate-buffered formaldehyde for 15 min at a filling pressure of 20 cmH_2_O. After embedding in paraffin, 4-μm-thick sections were cut, dewaxed, rehydrated and incubated in hematoxylin-eosin solutions (Carl Roth, Karlsruhe, Germany) to stain nuclei and tissue proteins. Lung elastic fibers were visualized by staining the sections with Weigert resorcin-fuchsin (0.05% in acid 70% ethanol; Waldeck, Münster, Germany) followed by counterstaining with tartrazine (0.25% in saturated picric acid; Sigma-Aldrich). Images of the stained sections were taken with the Axiovert microscope equipped with Spot Camera (Carl Zeiss, Jena, Germany). Lung histological parameters were analyzed by use of the ImageJ 1.47v software [21]. The mean linear intercept was determined according to the guidelines of Knudsen et al. [22]. The radial alveolar count was determined between most peripheral bronchiole and the nearest pleural surface according to Obayashi et al. [23].

### Expression analyses

Total RNA and protein was isolated from the right lung by use of the TRIzol™ Reagent (Thermo Fisher Scientific). In addition, we prepared tissue lysates in 1% phosphate-buffered sodium dodecyl sulfate solution supplemented with protease inhibitors. Equal protein amounts were separated by gel electrophoresis with subsequent standard immuno-blot procedure using polyclonal rabbit antibodies against SP-B (Santa Cruz, Santa Cruz, CA), SP-C (Santa Cruz), Cu/ZnSOD (Abcam, Cambridge, UK), catalase (Abcam), MnSOD (Rockland, Limerick, PA), PEX14 (Merck-Millipore, Darmstadt, Germany) or GAPDH (Santa Cruz). The expression of the antioxidant enzymes was determined by slot blot procedure using the Minifold slot blot system (GE Healthcare Life Science, Freiburg, Germany) with subsequent antibody detection. The LAS 3000 computer-based imaging system (FUJI Film, Tokyo, Japan) equipped with AIDA 2.0 software (Raytest, Straubenhardt, Germany) was used for signal detection and quantification of the signal intensities. All data were normalized per total protein stain of the blotted proteins, which was performed with 0.5% amido black solution.

Equal amounts of total RNA were reverse transcribed into cDNA with the M-MLV RT Rnase H Minus (Promega, Madison, WI). Then, the cDNA was subjected to quantitative PCR using the iCycler iQ™ Real-Time PCR Detection System (Rio-Rad, Hercules, CA). PCR was performed with mouse-specific primers for *SFTPB* (sense: 5′-agttctcctgctagacgcac-3′, anti-sense: 5′-ctgttcacacttttgcctgtc-3′), *SFTPC* (sense: 5′-tcctgatggagagtccaccg-3′, anti-sense: 5′-cagagcccctacaatcaccac-3′) or *ELN* (sense: 5′-tcctggagccactcttacag-3′, anti-sense: 5′-ctctctctccccaattagcc-3′). Relative mRNA amounts were quantified by use of an external cDNA standard because the normalization per another gene (*gapdh*, *selenbp1*, *tkt1* were tested as potential house-keeping genes) appeared to be inappropriate.

### ROS analysis

ROS are formed in mitochondria and to high extent also in other cellular compartments of lung samples [24]. Therefore, total cytoplasmic protein was isolated from lung tissue in ice-cold 4-(2-hydroxyethyl)-1-piperazineethanesulfonic acid buffer (5 μl∙mg^− 1^ wet tissue) containing 1 μg∙ml^− 1^ saponine (Sigma, Saint Louis, MO) as mild detergent and 50 μM deferoxamine mesylate (Sigma) as Fe^3+^ chelator. Nuclei were then removed by centrifugation (600 *g* for 10 min). 10 μl nuclei-free cytoplasmic fraction were mixed after 90 min (Fe^3+^ chelate effect completed) with 90 μl 1 mM 1-hydroxy-3-carboxy-2,2,5,5,-tetramethyl-pyrrolidine (CPH; Noxygen, Elzach, Germany) as a spin trap for O_2_^•-^. A computer-operated electron paramagnetic resonance spectrophotometer (MiniScope MS100; Magnettech, Berlin, Germany) equipped with MiniScope Control v2.7.3 analysis software (Microtech GmbH, Bad Kreuznach, Germany) measured the time-dependent formation of 3-carboxy-2,2,5,5-tetra-methyl-pyrrolin-1-oxyl (^•^CP) at room temperature every 10 min. O_2_^•-^ data were expressed as ^•^CP formation rate∙min^− 1^.

### Respiratory mechanics

The buffer-perfused mouse lung system from Hugo Sachs Elektronik-Harvard Apparatus (March-Hugstetten, Germany) analyzed the lung respiratory mechanics. We described this ex vivo method before in detail [25, 26]. In short, each mouse was placed in an artificial thoracic chamber and positive-pressure ventilated (90 breaths∙min^− 1^) via tracheal cannulation. Through cannulation of pulmonary artery and left atrium of the heart we ensured a constant perfusion flow of the lung vascular bed (1 ml∙min^− 1^ at 37 °C). RPMI 1640 medium (Thermo Fisher Scientific) was used as perfusion buffer and constantly purged with 5% CO_2_/95% N_2_ to yield pH 7.4 and O_2_-reduced conditions. Thereafter, the ventilation was switched to negative pressure ventilation with constant minimal pleural pressure (− 2 cmH_2_O) and specific maximal pleural pressures. Since the isolated lung is not surrounded by the pleurae, repeating augmented breaths every 3 min at − 20 cmH_2_O maximal pleural pressure stabilize the tidal volume (TV) that would otherwise decline steadily. The PULMODYN® Pulmonary Mechanics Data Acquisition software recorded the lung physiological parameters.

### Statistics

The software SigmaStat 3.5 and SigmaPlot 10 (Systat Software Inc., San Jose, CA) were used for statistical calculations and data presentations. The ANOVA test followed by Holm-Sidak method (parametric data) or ANOVA on Ranks followed by Dunn’s method (non-parametric data) was used for multiple comparisons. The Student’s t-test (parametric data) was used for comparing two groups. The survival of mice was tested for significance by log-rank test. *P* values ≤0.05 indicate a significant difference between the groups.

## Results

All mice of our study were treated with normoxia (N), moderate hyperoxia (mH) or severe hyperoxia (sH) until PND14. Expect for lung functional analysis in fully grown mice at PND120, most other parameters were analyzed earlier at PND60. Oxidative stress-related parameters were additionally studied in newborns and directly after treatment (PND14).

### General data

At PND60, the treatment of newborn mice with neonatal hyperoxia slightly reduced their survival rate which was significant for mice of the mH group and only a trend for those of the sH group (Table 1). This reduction was not associated with differences in physical development (body weight, lung weight) and physical activity in a running wheel (Table 1). However, mice of the mH group showed a slight increase of platelets in the whole blood and number of cells in the BAL fluid (Table 1). Several BAL parameters indicating inflammation and damage of the alveolo-capillary membrane remained unchanged (Table 1). We also did not identify changes of the soluble variant of the cell surface molecule RAGE (sRAGE), which is reduced in BAL samples following alveolar epithelial cell damage and lung emphysema [27].

**Table 1:** General parameters of PND60 mice treated with neonatal hyperoxia

Parameter		N		mH		sH	
		Normoxia		moderate Hyperoxia		severe Hyperoxia	
PND60-survival ^a^	(%)	80	80	68*	*	72	
Physical status							
body weight ^b^	(g)	19.3	± 2.80	19.4	± 2.40	19.2	± 2.90
wheel-running activity ^c^	(km·d^−1^)	7.32	± 1.71	7.68	± 2.23	7.62	± 2.38
Blood values							
erythrocytes ^b^	(n∙10^3^∙mm^−3^)	6.93	± 1.05	8.03	± 0.57	7.22	± 0.91
platelets ^b^	(n∙10^5^∙mm^−3^)	1.59	± 0.88	4.41	± 3.83*	2.31	± 2.11
leukocytes ^b^	(n∙10^3^∙mm^−3^)	9.78	± 2.53	9.65	± 3.79	9.68	± 2.78
Lung values							
lung-to-body weight ^b^	(·10^−3^)	1.29	± 0.28	1.28	± 0.26	1.37	± 0.28
lung wet-to-dry weight ^b^		8.32	± 1.43	7.84	± 1.66	8.80	± 1.38
BAL cells ^b, d^	(n·10^3^)	52.6	± 32.9	101	± 59.1*	58.2	± 40.7
BAL protein ^b^	(μg∙ml^−1^)	88.9	± 35.4	80.5	± 36.6	96.2	± 38.7
BAL IgM ^b^	(ng∙ml^−1^)	15.1	± 13.1	16.0	± 9.50	18.9	± 14.7
BAL sRAGE ^b^	(μg∙ml^−1^)	5.46	± 1.49	5.39	± 1.65	5.94	± 2.62

Data are means ± SD with *P < 0.05 vs. N group

^a^*n* = 80 in N group, *n* = 50 in mH group, *n* = 40 in sH group

^b^*n* ≥ 28 each group

^c^*n* = 17 each group. The respiratory function is more challenged by faster than slower running speeds. As female mice run faster and reach higher running distances than male mice [20], we only studied females

^d^Cytological investigations showed alveolar monocyte-like cells as major cell type (80%) followed by differentiated macrophages (19%), granulocytes (0.8%) and lung epithelial cells (0.2%). The relative quantity of these cell types was not altered in the mH or sH group

### ROS scavenger and free ROS

**Fig. 1:**
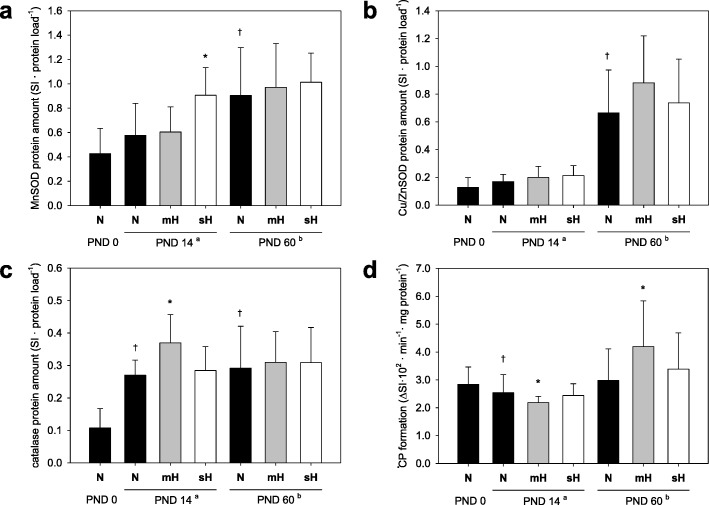
Antioxidant enzymes and ROS depending on age and neonatal hyperoxia. Protein amount of MnSOD (**a**), Cu/ZnSOD (**b**) and catalase (**c**) as well as level of endogenous ROS (^•^CP) formation (**d**) in mouse lung tissues depending on age and oxygen conditions. Mice were analyzed ^a^directly after finishing the oxygen treatment or ^b^later at PND60. Data are means ± SD (*n* ≥ 12 each group) with *P ≤ 0.05 vs. N of the same PND group and ^†^*P* ≤ 0.05 vs. PND0

Higher oxygen supply will increase the mitochondrial formation of O_2_^•-^ in mouse lung tissues [2]. For that reason, we quantified MnSOD and Cu/ZnSOD, which are important intra-cellular O_2_^•-^-scavenging enzymes, as well as catalase, which detoxifies resultant H_2_O_2_, in lung samples depending on development (age) and treatment. Development-dependent analyses showed increasing protein amounts of all three enzymes with early changes in catalase and late changes in MnSOD and Cu/ZnSOD (Fig. 1a-c). Hyperoxic conditions moderately increased the protein amounts of MnSOD in case of sH conditions (Fig. 1a) and catalase in case of mH conditions (Fig. 1c) when quantified directly after treatment (PND14) but not later. Detailed analysis of the O_2_^•-^ formation did not reveal a correlation of free O_2_^•-^ amount and the amount of selected O_2_^•-^-scavenging enzymes. Lung tissues of PND14 mice commonly formed less free O_2_^•-^, especially when they were treated with mH from birth (Fig. 1d). However, this effect was abolished later because lung tissues of PND60 mice of the mH treatment group generated more free O_2_^•-^ than those of the N or sH group (Fig. 1d).

### Lung histology and expression

**Fig. 2:**
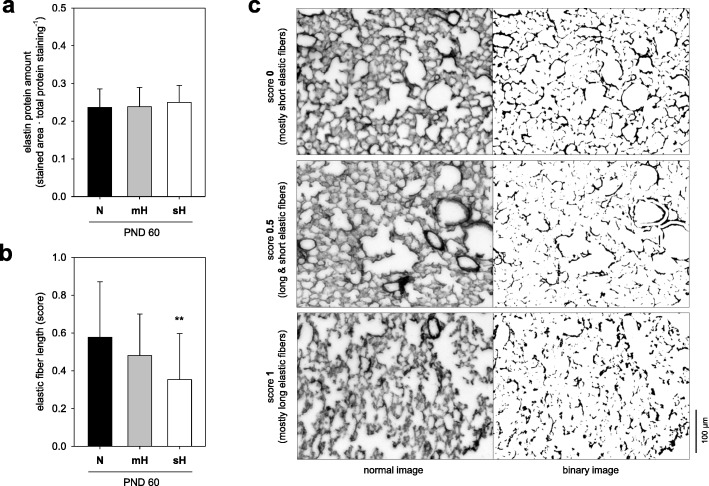
In situ elastin depending on neonatal hyperoxia. Lung analysis of PND60 treated with different concentrations of oxygen until PND14 for quantity (**a**) and quality (**b**) of elastin after staining of the lung sections with resorcin-fuchsin solution. Airways were omitted from the analysis. Elastin quality was assessed from the binary image by scoring the fiber lengths (**c**). Data are means ± SD (*n* = 24 each group) with ^**^P ≤ 0.01 vs. N group

Morphometric analyses of lung sections prepared from PND60 mice revealed significant changes in the lung structure of the sH but not mH treatment group. In this regard, we identified a lower alveolar density, thicker alveolar walls and augmented parenchymal airspace sizes with particular augmentation of the large airspaces (Table 2, Additional file 1: Figure S1). As result of these changes, less cell counts per tissue area but not per total area have been counted (Table 2). The amount of elastic fibers remained unchanged (Fig. 2a) but their quality was impaired as indicated by an increase in shortened fibers (Fig. 2b-c).

**Table 2:** Lung morphometric parameters of PND60 mice treated with neonatal hyperoxia

Parameter		N		mH		sH	
		Normoxia		moderate Hyperoxia		severe Hyperoxia	
Alveoli							
alveolar wall thickness	(μm)	12.3	± 2.90	13.9	± 5.35	17.6	± 5.41 ^***^
radial alveolar count	(n)	8.70	± 1.96	8.71	± 1.73	7.53	± 1.53 *
Cell number (stained nuclei)							
per tissue area ^a^	(n∙10^2^∙mm^−2^)	28.3	± 7.45	27.3	± 6.55	20.5	± 4.88 ^***^
per tissue and airspace area ^a^	(n∙10^2^∙mm^−2^)	1.55	± 2.80	1.53	± 2.95	1.41	± 1.96
Airspace size (mean linear intercept)							
all airspaces ^a^	(μm)	23.2	± 3.53	23.5	± 4.26	26.0	± 4.00 *
small airspaces ^b^	(μm)	11.7	± 2.89	12.3	± 3.14	13.2	± 2.18
medium airspaces ^b^	(μm)	17.1	± 2.96	17.9	± 3.45	19.5	± 3.21 *
large airspaces ^b^	(μm)	26.7	± 4.15	27.3	± 4.86	31.3	± 5.61 ^**^

All parameters were analyzed by use of hematoxylin-eosin-stained lung sections (see Additional file 1: Figure S1 for examples each treatment option)

Data are means ± SD (*n* = 24 each group) with *P ≤ 0.05, ^**^P ≤ 0.01 and ^***^P ≤ 0.001 vs. N group

^a^Areas of airways were omitted in order to get morphometric data of the lung parenchyma

^b^Sizing according to values of the mean linear intercept for lower quartile, median and upper quartile, respectively

**Fig. 3:**
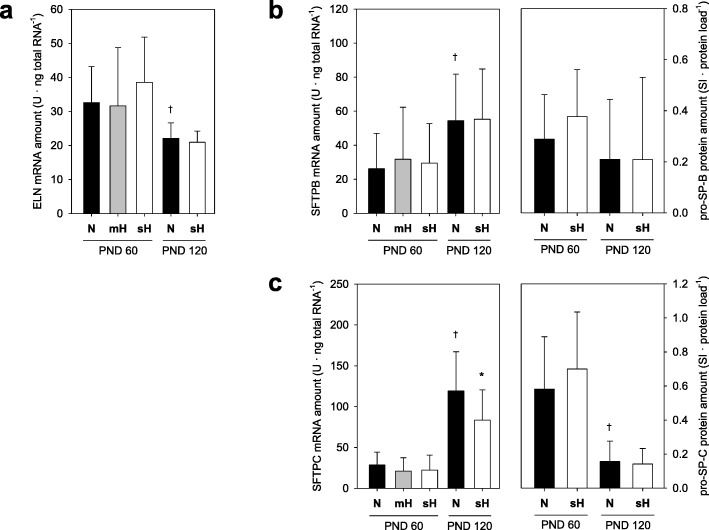
Expression of elastin and surfactant proteins depending on age and neonatal hyperoxia. Lung expression analyses of PND60 and PND120 mice treated with different concentrations of oxygen until PND14 indicate the mRNA amount of elastin (**a**) and the mRNA and protein amounts of the pro-surfactant proteins B (**b**) and C (**c**). Data are means ± SD (*n* ≥ 24 each group) with *P ≤ 0.05 vs. N of the same PND group and ^†^*P* ≤ 0.05 vs. N of the PND 60 group. SI, signal intensity; U, relative expression units per external standard curve

Functional elastin and pulmonary surfactant significantly contribute to the elasticity of the lung tissue. However, neonatal hyperoxia did also not influence the lung expression of elastin as indicated by unaltered mRNA amounts at PND60 (Fig. 3a). The mRNA and protein amounts of the pro-surfactant proteins (SPs) B and C were also not influenced by neonatal hyperoxia (Fig. 3b-c). In contrast, the lung expressions of elastin and both pro-SPs were significantly influenced by age. In this regard, the mRNA amount of elastin was reduced in fully grown mice at PND120 without influence of neonatal hyperoxia, which was analyzed for sH conditions only (Fig. 3a). The mRNA amounts of the pro-SPs B and C were increased with higher age but also not considerably altered by neonatal hyperoxia (Fig. 3b-c). Comparative analyses of mRNA and protein amount additionally revealed a reverse regulation of mRNA and protein depending on mouse age, in particular for pro-SP-C (Fig. 3b-c).

### Respiratory mechanics

**Fig. 4:**
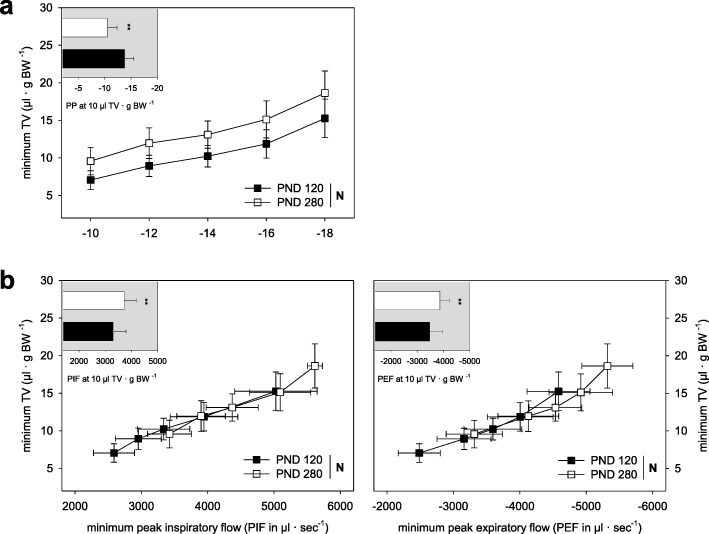
Ex vivo lung function depending on age. Respiratory mechanics of normoxic PND120 and PND280 mice recorded by use of a buffer-perfused isolated lung system. Pleural pressure-TV relations indicate the lung compliance (**a**). Peak airflow values are given at the pleural pressure-related TVs (**b**). For data presentation we used minimum values recorded shortly prior to the deep inspiration performed every 3 min and resultant values for the normal respiratory volume of mice (10 μl TV·g BW^− 1^). All data are means ± SD (*n* ≥ 12) with ^**^P ≤ 0.01 between the PND groups. BW, body weight; TV, tidal volume

**Fig. 5:**
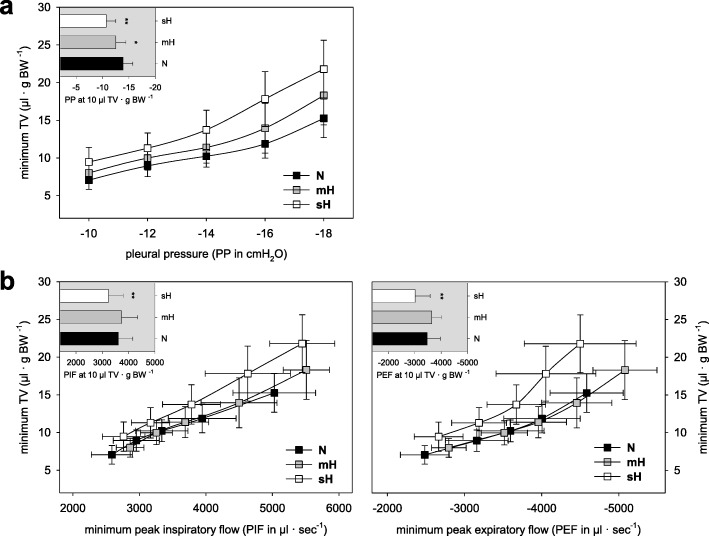
Ex vivo lung function after neonatal hyperoxia. Respiratory mechanics of PND120 mice treated with different concentrations of oxygen until PND14 were recorded by use of a buffer-perfused lung system. These parameters are the lung compliance presented by the pleural pressure-TV relations (**a**) and the peak airflow values recorded at the pleural pressure-related TVs (**b**). For data calculation we used minimum values recorded shortly prior to the deep inspiration performed every 3 min and resultant values for the normal respiratory volume of mice (10 μl TV·g BW^− 1^). The mean airway resistance at 70% TV (**c**) and the pulmonary artery pressures (**d**) as mean (left) and in dependence on changes in the perfusion flow (right) are given when the ventilation was performed at normal respiratory volume. Lung wet-to-dry ratio indicates the edema formation after lung ex vivo analysis (**e**). All data are means ± SD (*n* ≥ 16 each group) with *P ≤ 0.05 and ^**^P ≤ 0.01 vs. N group. BW, body weight; TV, tidal volume; PAP, pulmonary artery pressure per atmospheric pressure

Lung functional parameters were analyzed ex vivo in mice at PND120. In order to better assess potential lung functional changes caused by neonatal hyperoxia, we performed comparative analyses of untreated PND120 mice and older (PND280) mice in an initial experiment. This comparison revealed an age-related increase of the lung compliance as indicated by changes in the pleural pressure-TV relations (Fig. 4a) accompanied by higher peak airflows for inspiration and expiration (Fig. 4b). Like the untreated PND280 mice, PND120 mice treated with neonatal hyperoxia showed an increase of the lung compliance which was more pronounced in the sH than mH treatment group (Fig. 5a). Unlike the untreated PND280 mice, PND120 mice treated with neonatal hyperoxia did not show higher peak airflows for inspiration and expiration (Fig. 5b). In the case of neonatal sH condition, the peak airflows for inspiration and expiration were significantly reduced (Fig. 5b). Airway resistance and basal pulmonary artery pressure were not influenced by neonatal hyperoxia (Fig. 5d). Only the perfusion flow-dependent pulmonary artery pressure was slightly increased in PND120 mice of the sH group (Fig. 5d). Also, lungs of the sH group were more susceptible to edema formation due to our ex vivo ventilation procedure (Fig. 5e).

## Discussion

Our mouse experimental study demonstrated that neonatal hyperoxia at severe condition causes structural and functional changes of the adult lung indicating towards an emphysematous lung phenotype. This lung phenotype is not accompanied by oxidative stress and inflammation in adulthood. Also, it is not severe enough to restrict the relatively high running activity of mice. Assessments of the elastic fiber lengths and comparative analyses of the mRNA and protein level of elastin and selected surfactant proteins indicate a pivotal role of severe neonatal hyperoxia on long-lived extra-cellular matrix structures in adult lungs. In contrast to neonatal hyperoxia at severe condition, moderate hyperoxia does not cause such significant changes in lung histology and function in adulthood but, surprisingly, somewhat more lung oxidative stress. Moreover, direct comparisons of the respiratory mechanics of our young-adult mice treated with moderate neonatal hyperoxia with the respiratory mechanics of older untreated mice suggest potential functional impairment at advanced ages.

Computed tomography-based investigations of survivors of the severe BPD often show structural changes of the lung indicating lung emphysema [16, 17]. This clinical observation is in accordance with the histological changes, such as less alveolarization and enlarged parenchymal airspaces, we and others [10, 12, 28–31] observed in the lungs of adult mice exposed as newborns to severe hyperoxia (75–90% O_2_) for a comparable time period (10–14 d). The increased thickness of the alveolar walls and, therefore, lower cell number per total lung tissue area identified in our study might result from an aberrant expression of elastin and other extracellular matrix molecules [32]. In contrast to others, we additionally identified the particular augmentation of the larger parenchymal airspaces indicating a strong long-term effect on the alveolar ducts which are made of alveoli.

Clinical investigations of young-adult survivors of severe BPD also identified limitations in the expiration as indicated by lower values for FEV_1_ and FEF_25–75%_ [14–16], which are the functional consequences of emphysema-like histological changes in the lung. In accordance with these clinical observations, our functional analyses also identified limitations in the peak expiratory flows of mice treated with severe hyperoxia. This observation has not been made in other animal studies as they used other instruments, mainly plethysmographs [10–13, 33], for assessing the mouse respiratory function. In addition, our ex vivo analysis showed that lungs of mice treated with severe neonatal hyperoxia are more sensitive to ventilation-mediated damages. This could eventually become problematic in case a mechanical ventilation support is required later in life.

The higher sensitivity of the lung tissue to ventilation-mediated damages might be caused by the increased lung compliance together with lower expiratory flows because the volume of inspired air is critically high at higher pleural pressures. As isolated lungs are not restricted by the thoracic cage, this is also of particular case in our ex vivo lung analysis. In addition to differences in peak expiratory flow and lung compliance, severe neonatal hyperoxia causes reduced peak inspiratory flows without alterations in the airway resistance which suggests a general impairment of the lung elasticity in adulthood. Important reason for the lower lung elasticity might be the fragmentation of elastic fibers, which can be caused by an increase in neutrophile elastase during administered hyperoxia [34] and by an increased formation of extra-cellular ROS molecules via NADPH oxidases of the NOX family [35]. An ongoing fragmentation of elastic fibers weeks after the treatment with neonatal hyperoxia can be excluded because lung inflammatory processes (e.g. more BAL macrophages) or more ROS formation have not been observed in lung tissues of adult mice. As we did not identify lung inflammation in later life but elastin fragments act as matrikines for immune cells [36], it also suggests a failure of these elastin fragments to induce chemotaxis.

In contrast to the reduced length of elastic fibers, the total amounts of elastin as marker of fibroblasts [37], selected surfactant proteins as markers of type II alveolar epithelial cells [38] and the soluble variant of RAGE, a marker of type I alveolar epithelial cells [39], remained unchanged in adult mice treated with severe neonatal hyperoxia. This indicates a lower long-term importance of cellular than extra-cellular compounds. ROS-mediated damages of extra-cellular compounds, in particular the elastic fibers, are extremely critical because of their low turnover rates and, therefore, reduced replacement later in life [40]. This is demonstrated in an adult mouse model showing that the emphysema-related oxidative fragmentation of the lung extra-cellular matrix can be attenuated by overexpression of the extra-cellular SOD (EC-SOD) [41].

Our study also included analyses of selected antioxidant enzymes important for the removal of intracellular ROS. In accordance with most findings of Berkelhamer et al. [42], their amount increases in lung with postnatal age of mice. Surprisingly, neither catalase nor the intracellular SODs (MnSOD, Cu/ZnSOD) showed a clear up-regulation directly after hyperoxic conditions (PND14). An insufficient hyperoxia-mediated stimulation of antioxidant mechanisms is also suggested by the level of free ROS detected in lung. In case of more active antioxidant defense directly after hyperoxia, the endogenous level of free (absolute minus scavenged) ROS should have been significantly reduced at this time point. However, this was only observed to some extent in lung tissues of PND14 mice treated with moderate hyperoxia.

The data of study also showed that experimental hyperoxia using oxygen concentration of 75% until the completion of the saccular phase in mice (about PND15 [7]) is high enough to induce long-term damages of the lung which are similar to the clinical picture of the adult BPD [14–17]. However, these lung changes were not associated with a reduced physical exercise capacity of mice as expected from findings of clinical and experimental studies [5, 43]. One reason for this discrepancy could be that our study assessed the voluntary but not maximum exercise capacity. Another reason could be that the lung structural and functional changes induced by neonatal 75% oxygen are not severe enough to limit the physical exercise capacity. The later reason is supported by the fact that only animals treated with highly severe hyperoxia (95% O_2_ for 14 d) showed restrictions in their physical capability [43]. Highly severe hyperoxia also causes an increased airway resistance due to damages of the conducting airways [10, 12], an alteration which has not been observed in our lung functional analysis.

In clinical situations, the surfactant replacement may mediate significant antioxidant activity due to enzymatic and non-enzymatic compounds naturally present in animal surfactants [44]. This is of course very important for the treatment of the respiratory distress syndrome in preterm infants with high concentrations of supplementary oxygen because animal surfactants will partially scavenge ROS and, therefore, compensate for the oxidative damages of the elastic fibers and other proteins in lung tissues. Moreover, the surfactant therapy has led to the requirement of lower oxygen concentrations for treating the respiratory distress syndrome, but even moderate conditions of experimental hyperoxia (50% O_2_) cause changes of the respiratory mechanics in adulthood. In this regard, we identified higher lung compliance but without increase in the peak expiratory flow as expected from an older comparison group. This observation suggests that neonatal hyperoxia at moderate conditions may induce an age-related lung emphysema [45] earlier in life. However, the mice analyzed in our experimental study as well as most preterm infants participating as adults in the human studies were not old enough in order to estimate their age-related decline in the respiratory function [14–17].

Lung tissues of adult mice exposed as newborns to moderate hyperoxia also showed a slight increase in free ROS associated with somewhat more immune cells located in the BAL fluid. It is well conceivable that the NADPH-dependent generation of ROS in BAL cells contributes to an increased lung level in free ROS [46]. Although we do not know what might have caused more immune cells in the BAL fluid, the slight ROS increase is not necessarily of disadvantage because ROS essentially contribute as second messengers to healthy cell function [47]. Moreover, the blood cell count of adult mice treated with moderate neonatal hyperoxia differed from untreated mice with particular increase in platelets. Murine platelets are mainly generated from megakaryocytes in the lung tissue [48] and are able to support the lung development [49]. Therefore, its increase could suggest higher activity of cellular processes compensating for the oxygen-mediated damages, but the potential role of platelets in the reduction of oxygen-mediated damages in newborn lungs is still unknown.

As already discussed above, our animal experimental study using newborn mice identified some similarities to the hyperoxia-mediated lung pathologies described in human survivors of the preterm birth. The existence of such similarities indicates the usefulness of this mouse model to study the effect of neonatal hyperoxia on lung structure and function in adulthood. However, the lung of newborn mice is not that premature (late saccular stage) as the lung of most premature human infants requiring the treatment with high concentrations of oxygen. Also, newborn mice are adapted to survive with a lung in the late saccular stage without supplemental oxygen. Therefore, lung tissues of human preterm infants may be more susceptible to the same oxygen concentrations used in our animal study.

## Conclusions

Moderate and severe hyperoxic conditions in newborn mice cause differential lung phenotypes in adulthood. Emphysematous lungs without persisting oxidative stress and inflammation are the result of severe neonatal hyperoxia. As the existing fragmentation of the elastic fibers seems to play a pivotal role in the development of this lung phenotype, more effort should be made to identify useful elastin-protecting compounds which can be applied in addition to surfactant therapy in the future. Non-emphysematous lungs with slight increase in ROS formation and some other changes are the result of moderate neonatal hyperoxia. However, detailed experimental studies are still needed in order to understand their possible importance in the compensation of hyperoxic lung damages.

## Supplementary information

**Additional file 1: Figure S1.** Altered lung structure in response to neonatal hyperoxia.

## Data Availability

Datasets generated during the current study are available from the corresponding author on reasonable request.

## Abbreviations

BAL: Bronchoalveolar lavage
BCA: Bicinchoninic acid
BND: Postnatal day
BPD: Bronchopulmonary dysplasia
BW: Body weight
CP: 3-carboxy-2,2,5,5-tetra-methyl-pyrrolin-1-oxyl
CPH: 1-hydroxy-3-carboxy-2,2,5,5,-tetramethyl-pyrrolidine
EC-SOD: Extra-cellular superoxide dismutase
FEF_x %_: Forced expiratory flow rates at x % vital capacity
FEV_1_: Forced expiratory volumes in 1 s
mH: Moderate hyperoxia
N: Normoxia
NADPH: Nicotinamide adenine dinucleotide phosphate
NOX: Nicotinamide adenine dinucleotide phosphate oxidase
PAP: Pulmonary artery pressure
PEF: Peak expiratory flow
PEX: Peroxisome biogenesis factor
PIF: Peak inspiratory flow
qPCR: Quantitative polymerase chain reaction
RAGE: Receptor for advanced glycation end-products
ROS: Reactive oxygen species
sH: Severe hyperoxia
SI: Signal intensity
SOD: Superoxide dismutase
SP: Surfactant protein
sRAGE: Soluble receptor for advanced glycation end-products
TV: Tidal volume
U: Expression units
