# Correction to: Severe but not moderate hyperoxia of newborn mice causes an emphysematous lung phenotype in adulthood without persisting oxidative stress and inflammation

**DOI:** 10.1186/s12890-020-1051-z

**Published:** 2020-01-21

**Authors:** Anke Kindermann, Leonore Binder, Jan Baier, Beate Gündel, Andreas Simm, Roland Haase, Babett Bartling

**Affiliations:** 1 Department of Cardiac Surgery, Middle German Heart Center, University Hospital Halle (Saale), Martin Luther University Halle-Wittenberg, Ernst-Grube-Str. 40, 06120 Halle (Saale), Germany; 2 Department of Neonatology and Pediatric Intensive Care, Clinic for Child and Adolescent Medicine, University Hospital Halle (Saale), Martin Luther University Halle-Wittenberg, Halle (Saale), Germany

**Correction to: BMC Pulm Med**

**https://doi.org/10.1186/s12890-019-0993-5**

Following publication of the original article [1], the authors flagged that the article had published with an error in ‘Table 1’.

The error was that in the row **PND60-survival**^**a**^ the value ‘80’ was erroneously repeated, and the special symbol (*) contained in the value ‘68*’ was erroneously repeated after the value.

Table 1 has now been corrected in the published article.

Please find the corrected Table 1 below for reference.

**Table 1:** General parameters of PND60 mice treated with neonatal hyperoxia

Parameter		N		mH		sH	
		Normoxia		moderate Hyperoxia		severe Hyperoxia	
PND60-survival ^a^	(%)	80		68^*^		72	
Physical status							
body weight ^b^	(g)	19.3	± 2.80	19.4	± 2.40	19.2	± 2.90
wheel-running activity ^c^	(km·d^− 1^)	7.32	± 1.71	7.68	± 2.23	7.62	± 2.38
Blood values							
erythrocytes ^b^	(n∙10^3^∙mm^−3^)	6.93	± 1.05	8.03	± 0.57	7.22	± 0.91
platelets ^b^	(n∙10^5^∙mm^−3^)	1.59	± 0.88	4.41	± 3.83^*^	2.31	± 2.11
leukocytes ^b^	(n∙10^3^∙mm^−3^)	9.78	± 2.53	9.65	± 3.79	9.68	± 2.78
Lung values							
lung-to-body weight ^b^	(·10^−3^)	1.29	± 0.28	1.28	± 0.26	1.37	± 0.28
lung wet-to-dry weight ^b^		8.32	± 1.43	7.84	± 1.66	8.80	± 1.38
BAL cells ^b, d^	(n·10^3^)	52.6	± 32.9	101	± 59.1^*^	58.2	± 40.7
BAL protein ^b^	(μg∙ml^−1^)	88.9	± 35.4	80.5	± 36.6	96.2	± 38.7
BAL IgM ^b^	(ng∙ml^−1^)	15.1	± 13.1	16.0	± 9.50	18.9	± 14.7
BAL sRAGE ^b^	(μg∙ml^−1^)	5.46	± 1.49	5.39	± 1.65	5.94	± 2.62

Data are means ± SD with *P < 0.05 vs. N group

^a^*n* = 80 in N group, *n* = 50 in mH group, *n* = 40 in sH group

^b^*n* ≥ 28 each group

^c^*n* = 17 each group. The respiratory function is more challenged by faster than slower running speeds. As female mice run faster and reach higher running distances than male mice [20], we only studied females

^d^Cytological investigations showed alveolar monocyte-like cells as major cell type (80%) followed by differentiated macrophages (19%), granulocytes (0.8%) and lung epithelial cells (0.2%). The relative quantity of these cell types was not altered in the mH or sH group
