# Supplementary material for: Severe but not moderate hyperoxia of newborn mice causes an emphysematous lung phenotype in adulthood without persisting oxidative stress and inflammation
Source: BMC Pulm Med. 2019 Dec 16;19:245. doi: 10.1186/s12890-019-0993-5 (PMC6915952; doi:10.1186/s12890-019-0993-5)
Supplement: Supplementary file 1 — Additional file 1: Figure S1. Altered lung structure in response to neonatal hyperoxia. [file 12890_2019_993_MOESM1_ESM.pptx]

## Slide 1
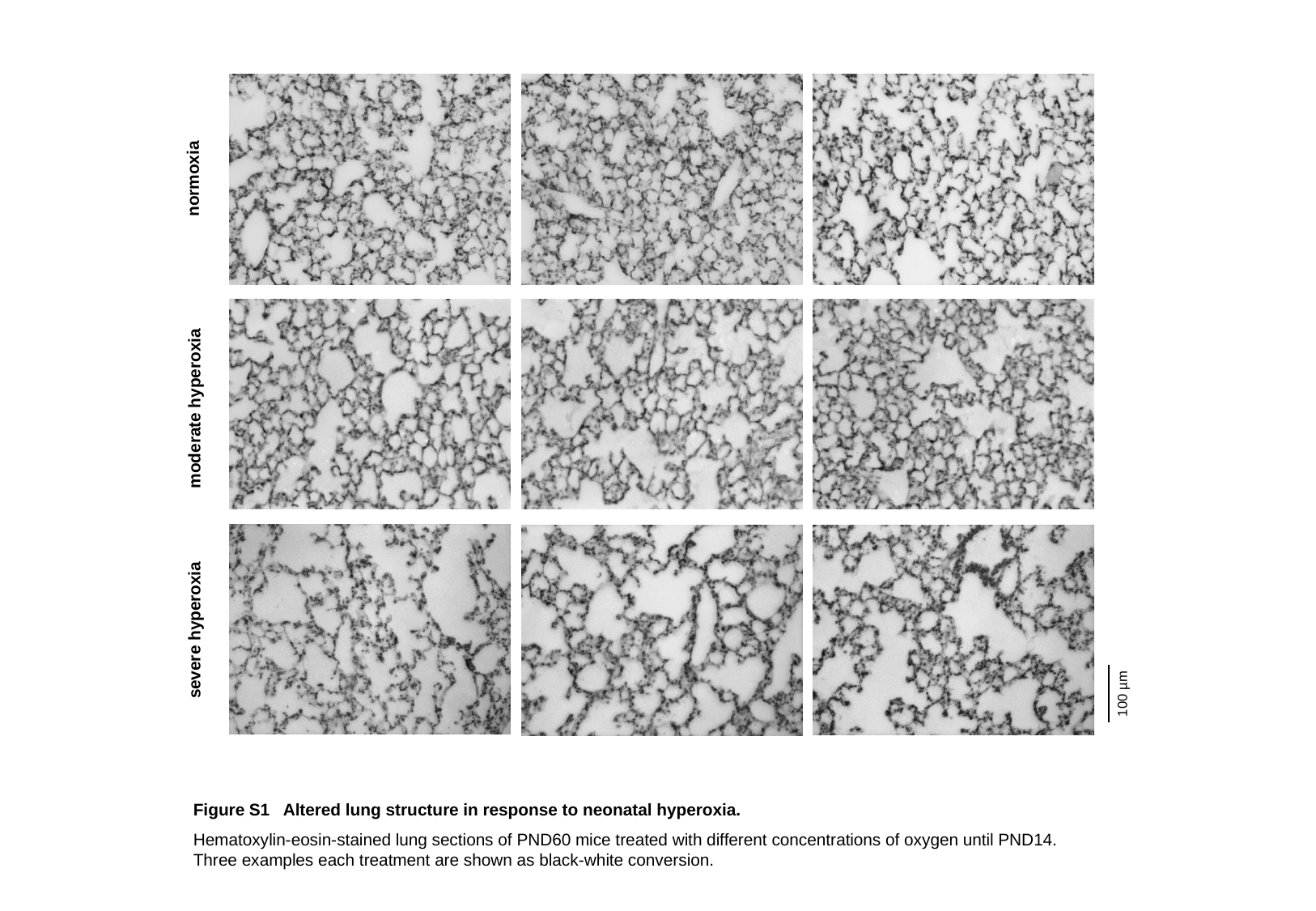

normoxia
moderate hyperoxia
severe hyperoxia
100 µm
Figure S1 Altered lung structure in response to neonatal hyperoxia.
Hematoxylin-eosin-stained lung sections of PND60 mice treated with different concentrations of oxygen until PND14.
Three examples each treatment are shown as black-white conversion.
